# Supplementary material for: Protein phosphatase 2A inactivation induces microsatellite instability, neoantigen production and immune response
Source: Nat Commun. 2021 Dec 15;12:7297. doi: 10.1038/s41467-021-27620-x (PMC8674339; doi:10.1038/s41467-021-27620-x)
Supplement: Supplementary file 1 — Supplementary Information [file 41467_2021_27620_MOESM1_ESM.pdf]

Supplementary Figures:

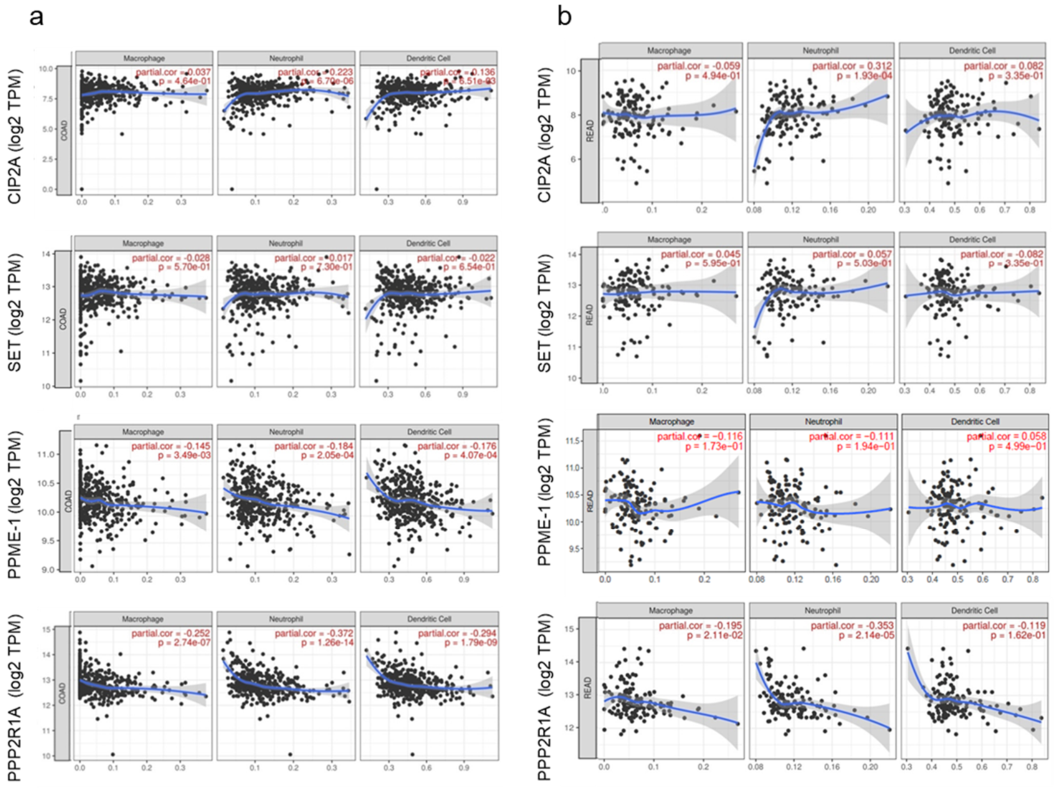

Supplementary Figure 1. Correlation of CIP2A, SET and PPP2R1A expression with neutrophils, macrophages and dendritic cell in (a) TCGA-COAD (n=461) and **(b)** TCGA-READ (n=172) dataset. Results are determined by Spearman correlation.

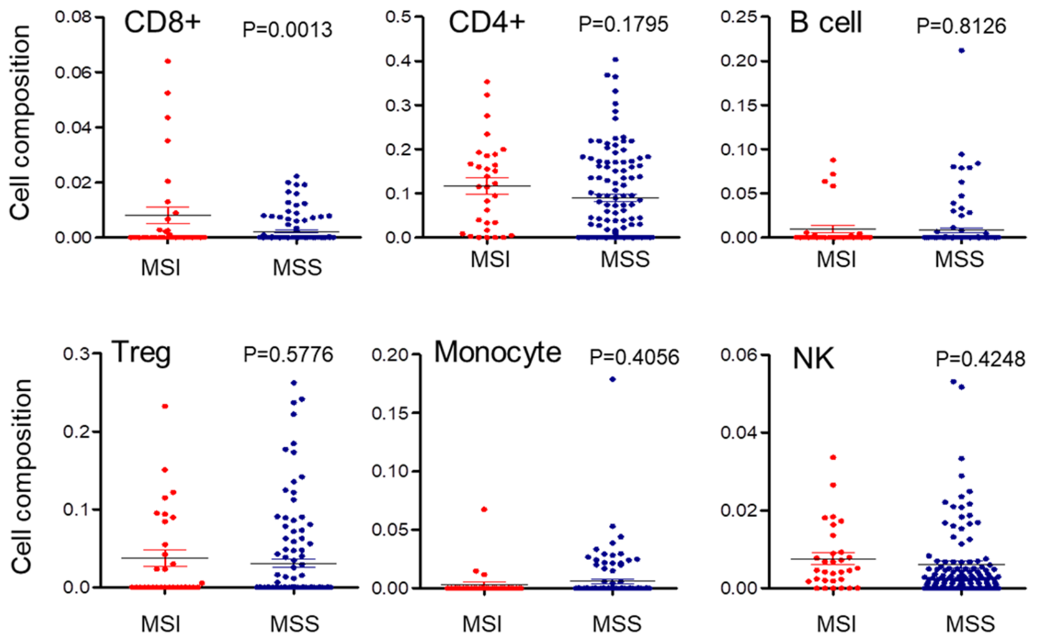

Supplementary Figure 2. MSI tumours possess higher infiltration of cytotoxic T cells. Analysis of mRNA level data, extracted from the TCGA-COAD dataset (n=144 for each) revealed higher composition of CD8+ T effector gene signatures in MSI compared to MSS. Data presented as mean ± s.e.m. with P value determined by two-sided unpaired t-test.

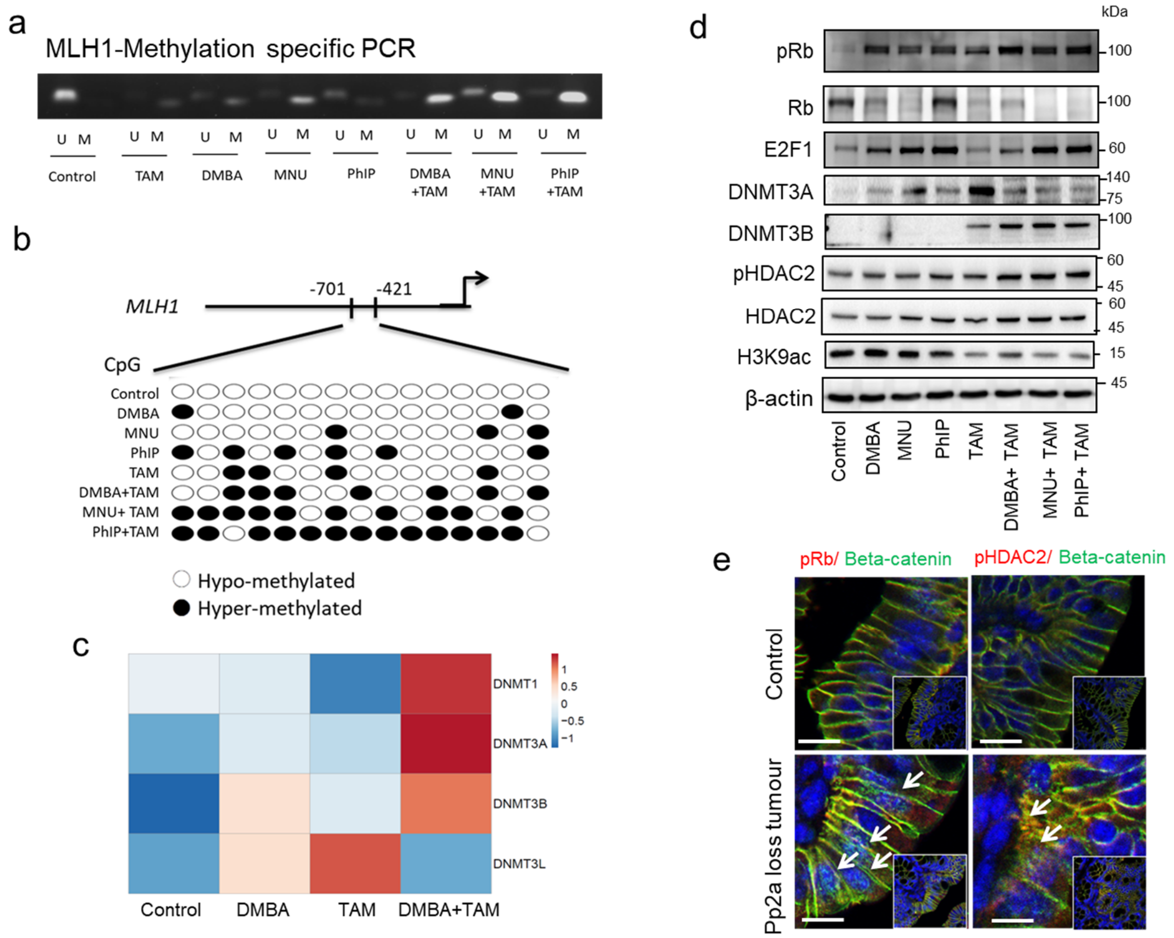

Supplementary Figure 3. MLH1 downregulation caused by Ppp2r1a deletion associates with promoter DNA hypermethylation and DNMT3A/B upregulation.
Lgr5-EGFP-CreERT2; Ppp2r1a$^{flox/flox}$ intestinal organoids were treated with DMBA, MNU, or PhIP in combination with or without tamoxifen (TAM) for 50 days. Hypermethylation of the mMLH1 promoter region CpG island in mouse intestine organoid cultures treated with each carcinogen and TAM. **(a)** Methylation-specific PCR of mMLH1. U, fragments amplified by unmethylated sequence-specific primers; M, fragments amplified by methylated sequence-specific primers. Image is representative of two biological independent samples for each group. **(b)** Bisulphite sequencing analysis of the endogenous MLH1 promoter. (Top) Schematic of the mMLH1 promoter; positions of CpGs are shown to scale by vertical lines. (Bottom) Each circle represents a methylated (black) or unmethylated (white) CpG dinucleotide. **(c)** Heat map of differentially expressed genes associated with DNA methyltransferase enzymes gene. **(d)** Western blot analysis of organoid cultures treated with indicated conditions. Blots are representative of three biological independent samples for each group. **(e)** Ppp2r1a-loss colon tumours were induced in *Lgr5-EGFP-CreERT2; Ppp2r1a*$^{flox/flox}$ by treatment with DMBA and TAM for 36 days. Control colon tissues (Control) and Ppp2r1a-loss colon tumours were harvested for analysis. Representative images of three biological independent immunofluorescence for each group showing increased p-Rb and p-HDAC2 levels in murine Ppp2r1a-loss colon tumours compared to controls. Arrows indicate double-positive signals. bar=25 μm. Source data are provided as a Source Data file.

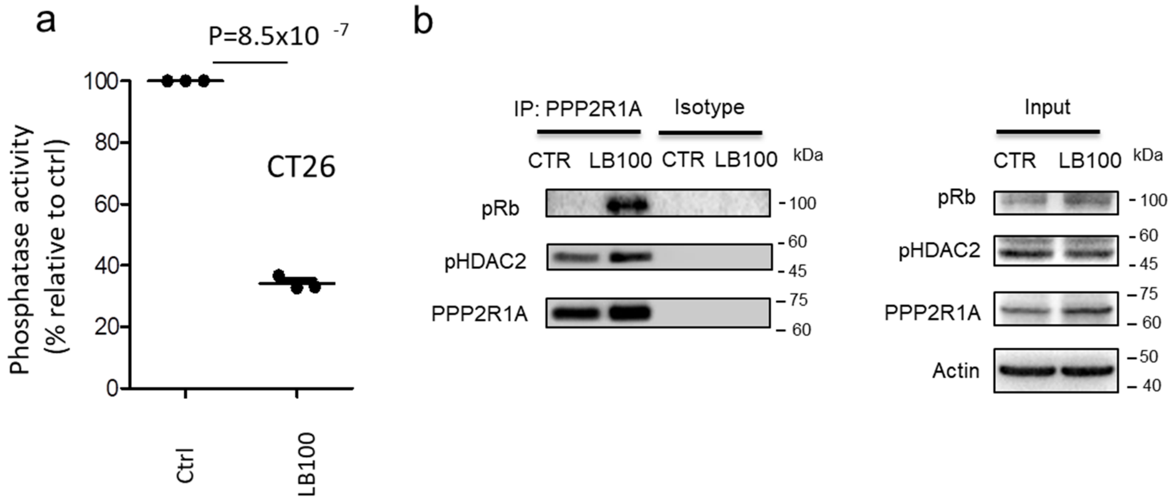

Supplementary Figure 4. Demonstration of PP2A activity inhibited by LB100 and interaction of PPP2R1A with Rb and HDAC2. CT26 cells were treated with 2.5 μM LB100 for 24 h (n=3), followed by PPP2R1A immunoprecipitation for **(a)** analysis of PP2A activity and **(b)** western blotting of phospho-Rb and phospho-HDAC2. Mean ± s.d. of three independent experiments with P value determined by two-sided unpaired t-test. Blots are representative of two biological independent samples for each group. Source data are provided as a Source Data file.

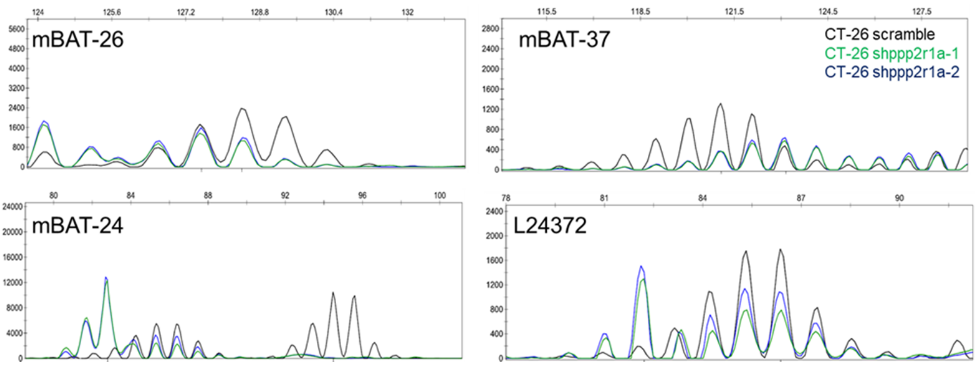

Supplementary Figure 5. PPP2R1A knockdown reprograms MSS toward MSI

The MSI status was evaluated by comparing mononucleotide repeats of CT26 transfected with scramble, ppp2r1a shRNA #1 (shppp2r1a-1) and #2 (shppp2r1a-2). The mononucleotide regions mBAT-26, mBAT-37, mBAT-24 and L24372 were used to evaluate microsatellite instability. MSI images are representative of two biological independent samples for each group.

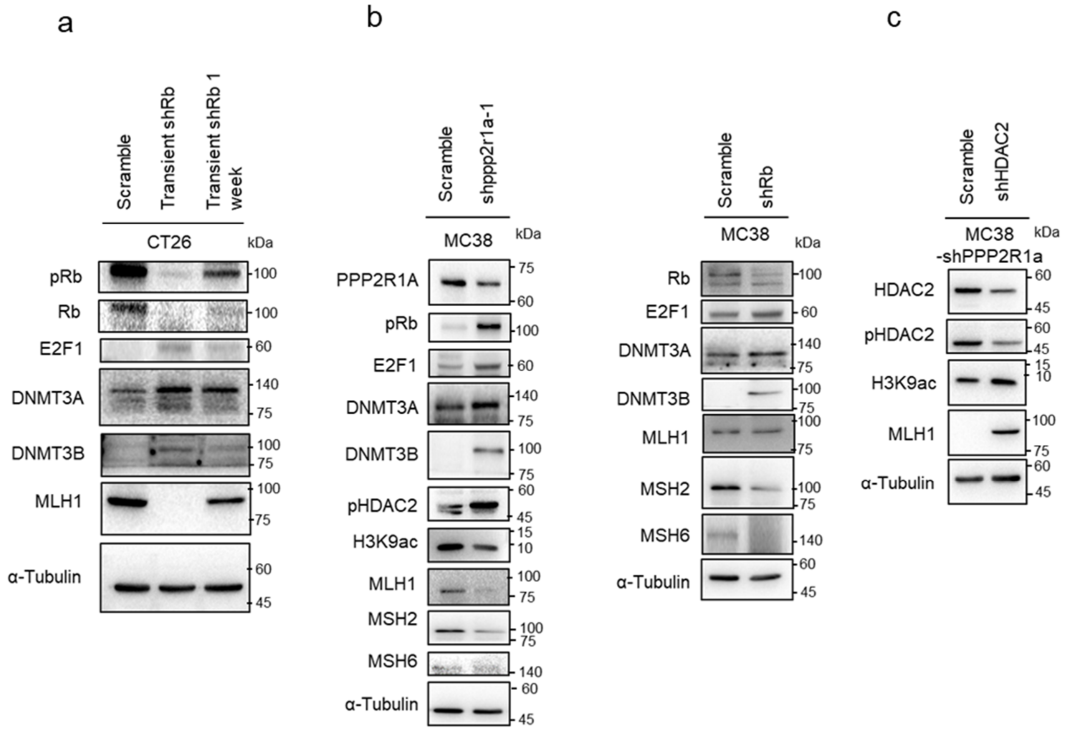

Supplementary Figure 6. Effects of Rb or HDAC2 knockdown in mouse colon MSS and MSI cell lines. Western blot analysis of **(a)** CT26 transiently transfected with scrambled or Rb shRNA (shRb). **(b)** MC38 transfected with scramble, ppp2r1a shRNA #1 (shppp2r1a-1), Rb shRNA (shRb) or **(c)** HDAC2 shRNA (shHDAC2). Blots are representative of two biological independent samples for each group. Source data are provided as a Source Data file.

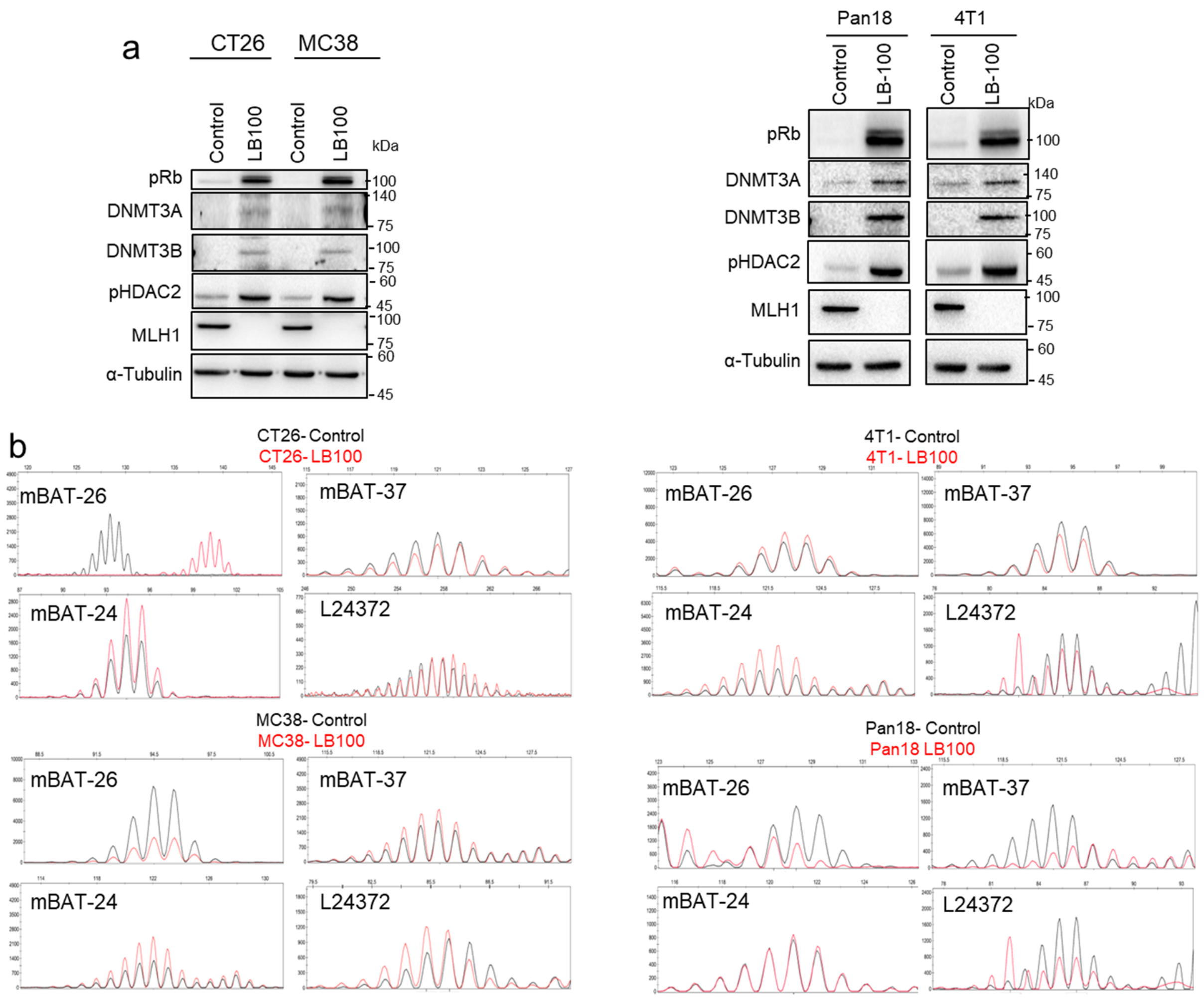

Supplementary Figure 7. PP2A inhibition reduces MLH1 level and induces MSI status. **(a)** Western blot analysis of the indicated CT26, MC38, 4T1 and Pan18 treated with vehicle control or PP2A inhibitor, LB100 (2.5 µM) for 2 days. **(b)** The MSI status was evaluated by comparing mononucleotide repeats of each corresponding cell. The mononucleotide regions mBAT-26, mBAT-37, mBAT-24 and L24372 were used to evaluate microsatellite instability. Blots and MSI status are representative of two biological independent samples for each group. Source data are provided as a Source Data file.

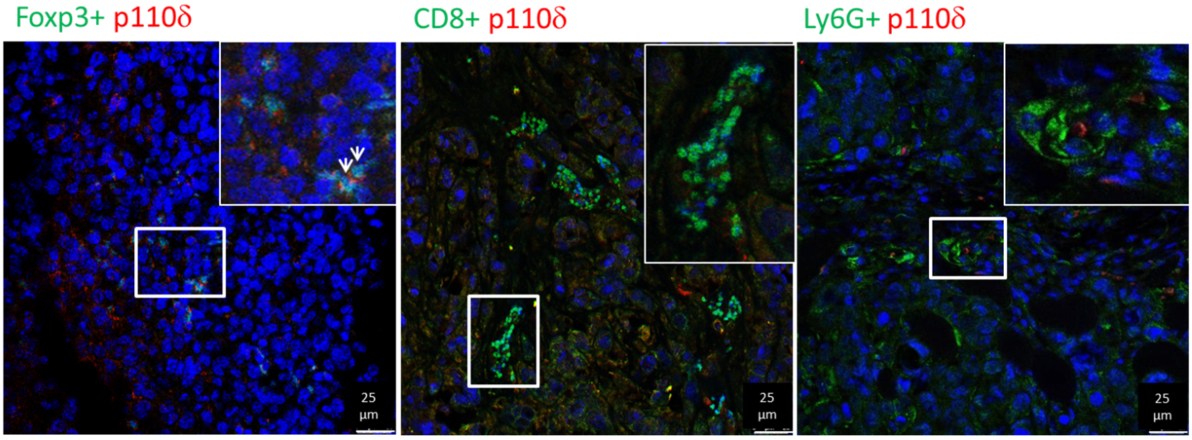

Supplementary Figure 8. Representative images showing colocalization of p110δ with Treg maker Foxp3 but not with CD8 or Ly6G. Specimens of CT26 tumour were subjected to double immunofluorescence of p110δ (red), and each marker of Treg (left panel, Foxp3+, green), CD8+ (centre panel, green) and PMN-MDSCs (right panel, Ly6Ghigh, green). Tumour sections were counterstained with DAPI for staining nuclei. Magnified pictures are shown in right upper corner. Images are representative of three biological independent samples for each group. Arrows indicate cells double positive for p110δ and Foxp3. bar=25 μm..

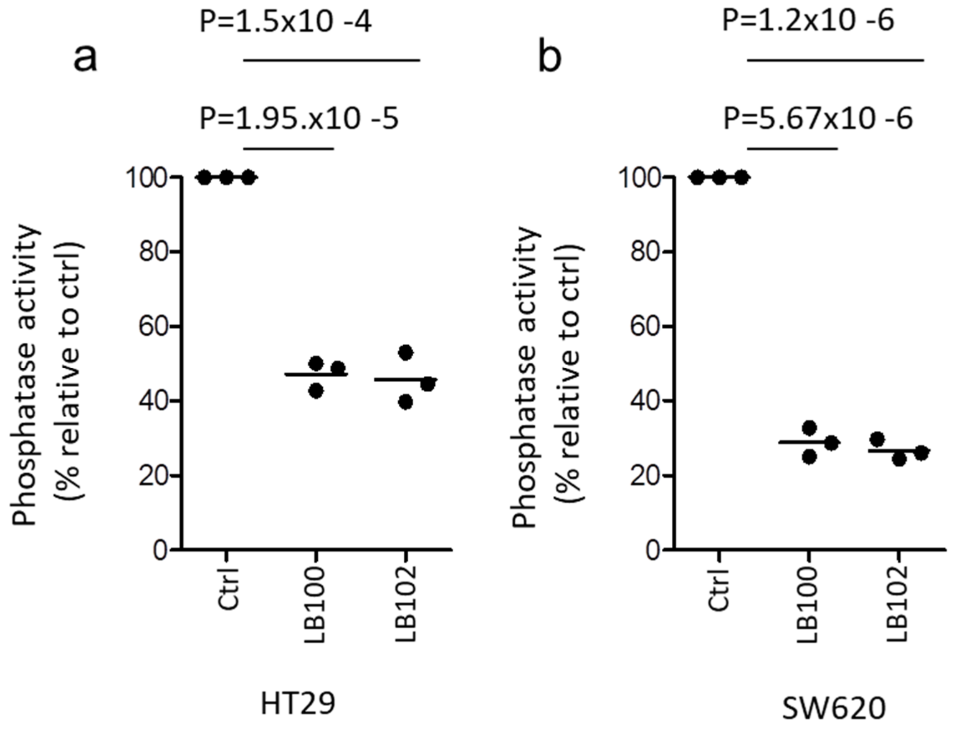

Supplementary Figure 9. LB100 and LB102 inhibit PP2A phosphatase activity. (a) HT29 and **(b)** SW620 cells were treated with 2.5 μM LB100 or 2.5 μM LB102 for 24h (n=3), followed by immunoprecipitation of cell lysates with anti-PP2A antibody. The immunoprecipitants were subjected to PP2A phosphatase activity measurement. Mean ± s.d. of three independent experiments with P value determined by two-sided unpaired t-test.

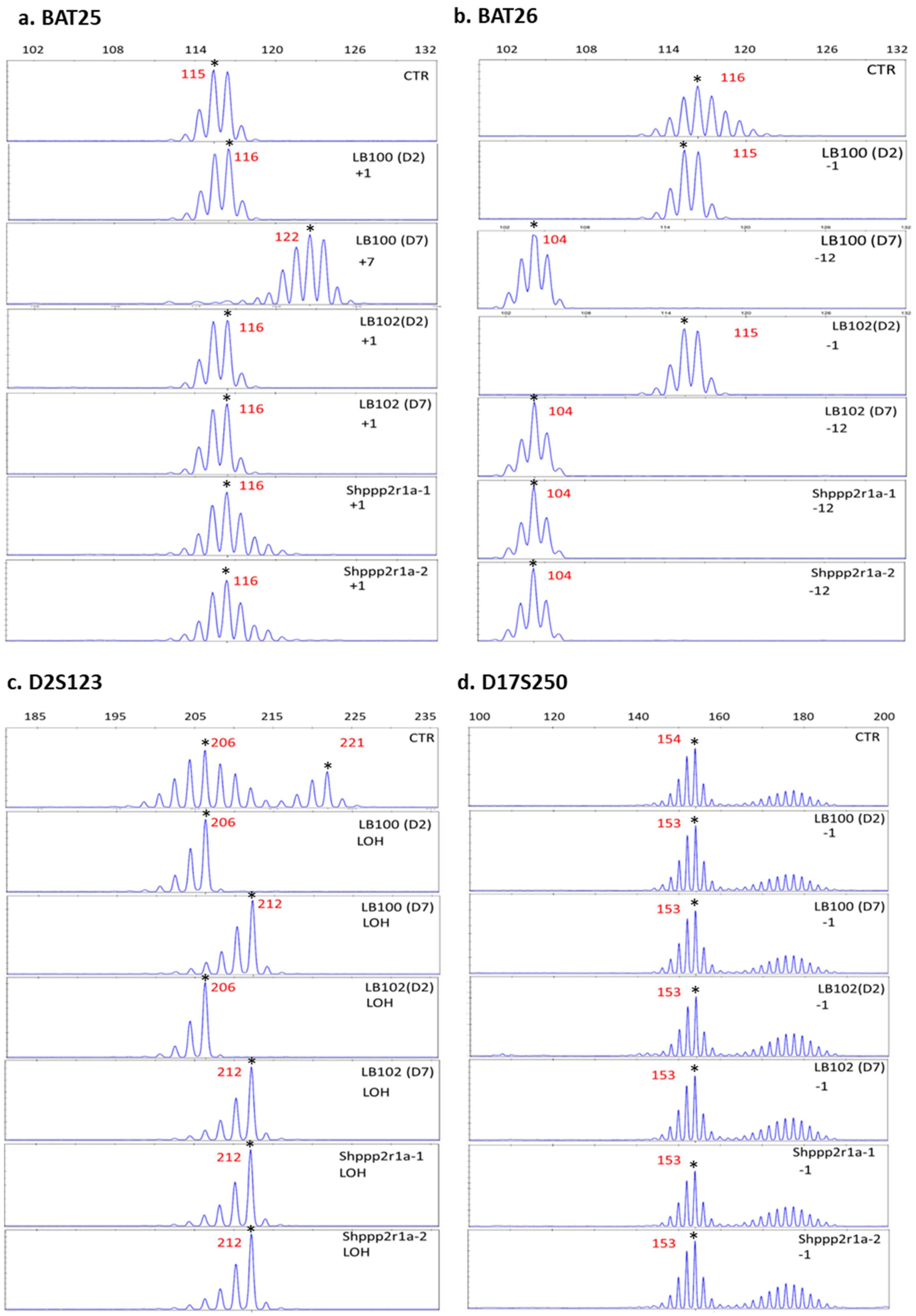
a. BAT25
102 108 114 120 126 132
115 CTR
116 LB100 (D2) +1
122 LB100 (D7) +7
116 LB102(D2) +1
116 LB102 (D7) +1
116 Shppp2r1a-1 +1
116 Shppp2r1a-2 +1
b. BAT26
102 108 114 120 126 132
116 CTR
115 LB100 (D2) -1
104 LB100 (D7) -12
115 LB102(D2) -1
104 LB102 (D7) -12
104 Shppp2r1a-1 -12
104 Shppp2r1a-2 -12
c. D2S123
185 195 205 215 225 235
206 221 CTR
206 LB100 (D2) LOH
212 LB100 (D7) LOH
206 LB102(D2) LOH
212 LB102 (D7) LOH
212 Shppp2r1a-1 LOH
212 Shppp2r1a-2 LOH
d. D17S250
100 120 140 160 180 200
154 CTR
153 LB100 (D2) -1
153 LB100 (D7) -1
153 LB102(D2) -1
153 LB102 (D7) -1
153 Shppp2r1a-1 -1
153 Shppp2r1a-2 -1

e. D5S346

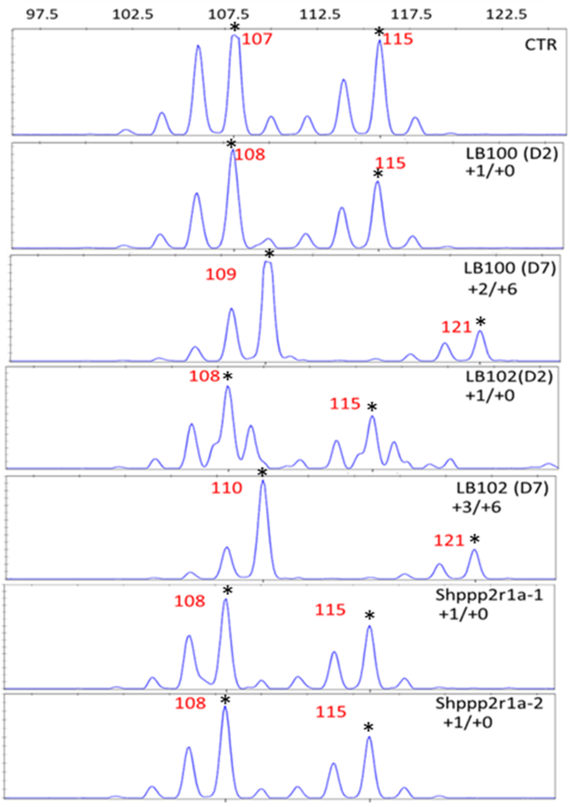

Supplementary Figure 10. Detection of MSI by analysis of microsatellite markers in HT-29 control and indicated treated cells. The treatments include LB100 and LB102 for 2 days (D2) and 7 days (D7), ppp2r1a knockdown with different shRNAs (Shppp2r1a-1, Shppp2r1a-2). The predominant amplicon size bands (peaks) in mutant alleles are indicated as red number(s) and asterisk(s) in all sample traces. Shiftings comparing to control (CTR) are marked at right under each sample name. The mononucleotide regions were used to evaluate microsatellite instability as follows: **(a)** BAT25, **(b)** BAT26, **(c)** D2S123, **(d)** D17S250 and **(e)** D5S346. MSI images are representative of two biological independent samples for each group.

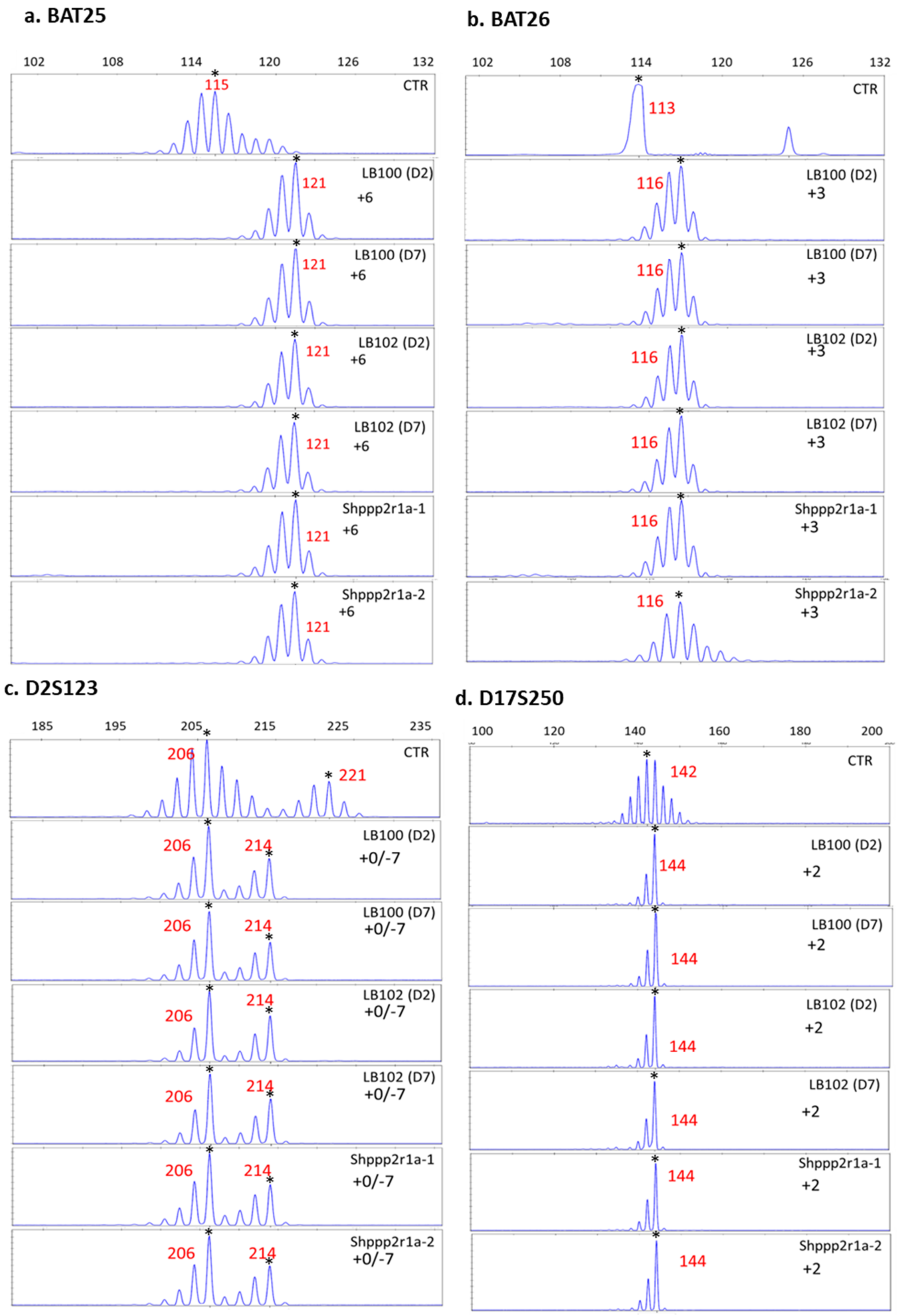
a. BAT25
102
108
114
120
126
132
115
CTR
121
LB100 (D2)
+6
LB100 (D7)
LB102 (D2)
LB102 (D7)
Shppp2r1a-1
Shppp2r1a-2
b. BAT26
113
116
+3
c. D2S123
185
195
205
215
225
235
206
221
214
+0/-7
d. D17S250
100
120
140
160
180
200
142
144
+2

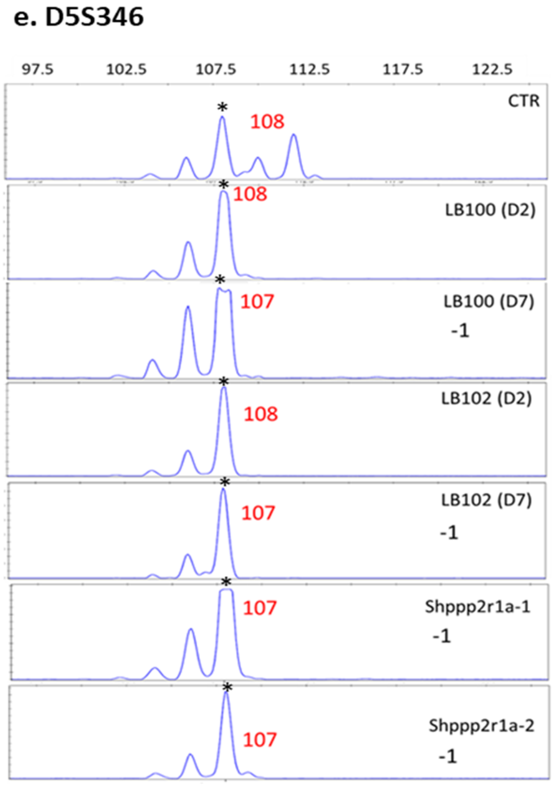

Supplementary Figure 11. Detection of MSI by analysis of microsatellite markers in SW620 control and indicated treated cells. The treatments include LB100 and LB102 for 2 days (D2) and 7 days (D7), ppp2r1a knockdown with different shRNAs (Shppp2r1a-1, Shppp2r1a-2). The predominant amplicon size bands (peaks) in mutant alleles are indicated as red number(s) and asterisk(s) in all sample traces. Shiftings comparing to control (CTR) are marked at right under each sample name. The mononucleotide regions were used to evaluate microsatellite instability as follows: **(a)** BAT25, **(b)** BAT26, **(c)** D2S123, **(d)** D17S250 and **(e)** D5S346. MSI images are representative of two biological independent samples for each group.

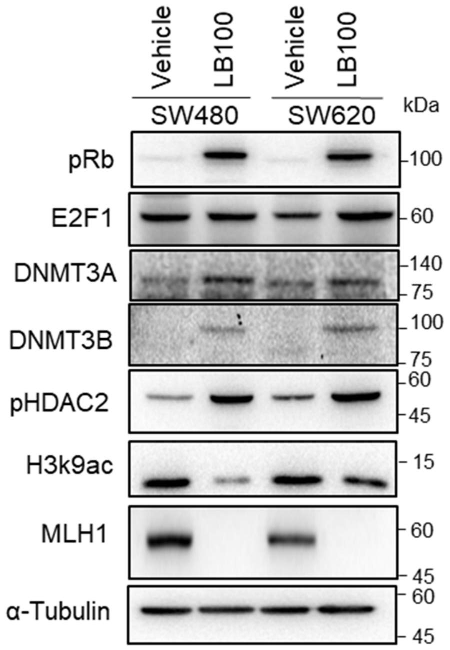

Supplementary Figure 12. PP2A inhibition reduces MLH1 level. Western blot analysis of the indicated SW480 and SW620 treated with vehicle control or PP2A inhibitor, LB100 (2.5 μM), for 2 days. Blots are representative of two biological independent samples for each group. Source data are provided as a Source Data file.

a

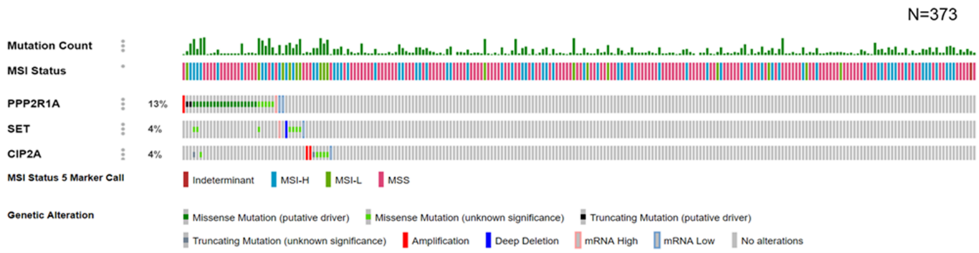

b

Clinical Attribute	Statistical Test	p-Value	q-Value
Mutation Rate Cluster	Chi-squared Test	1.09E-09	2.41E-08
MSI Status 5 Marker Call	Chi-squared Test	1.49E-03	7.11E-03
Cancer Type Detailed	Chi-squared Test	1.94E-03	7.11E-03
Mutation Count	Kruskal Wallis Test	0.0118	0.0289

Supplementary Figure 13. Analysis of genetic alteration of PPP2R1A, SET and CIP2A and MSI status in TCGA-uterine corpus endometrial carcinoma (n=373). (a) PPP2R1A, SET and CIP2A gene expression and mutation analysis; **(b)** Comparison of clinical attributes between cases with and without genetic alteration are determined by Chi-squared test and Kruskal Wallis test.

a

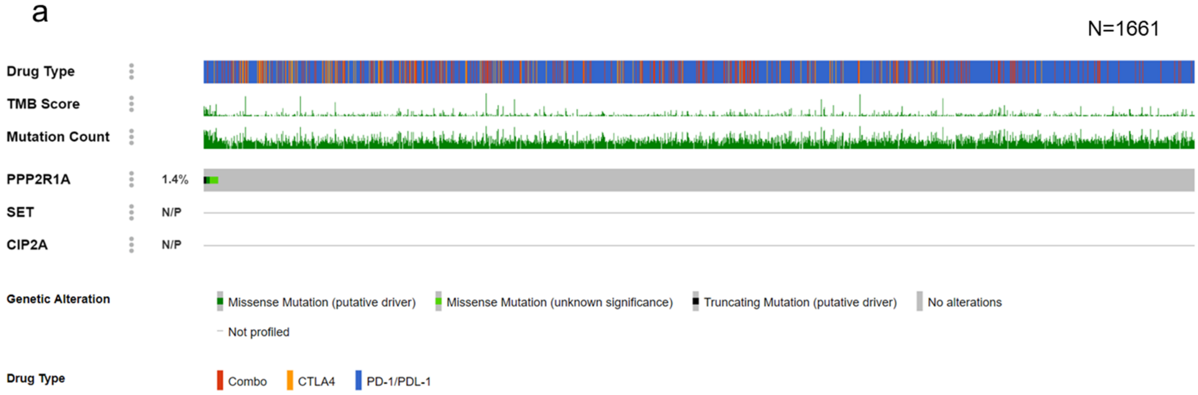

b

Clinical Attribute	Statistical Test	p-Value	q-Value
Metastatic Site	Chi-squared Test	7.80E-09	1.18E-07
TMB Score	Kruskal Wallis Test	8.10E-08	3.65E-07
Mutation Count	Kruskal Wallis Test	8.12E-08	3.65E-07
Overall Survival Status	Chi-squared Test	0.0232	0.0834

c

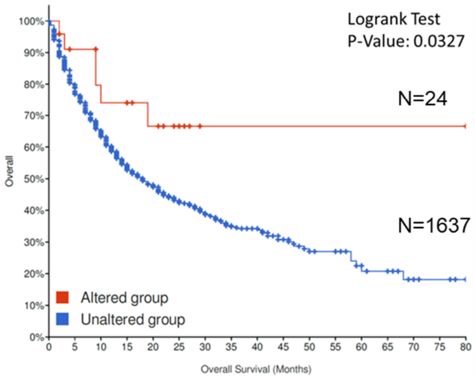

Supplementary Figure 14. Analysis of genetic alteration of PPP2R1A, SET and CIP2A in MSK-IMPACT (n=1661). (a) PPP2R1A, SET and CIP2A mutation analysis; **(b)** Comparison of clinical attributes between cases with and without genetic alteration. **(c)** Kaplan-Meier survival analysis of MSK-IMPACT patients stratified by altered and unaltered groups. Comparing with patients with PPP2R1A-nonmutated tumors, patients with PPP2R1A-mutated tumors had significantly lower overall mortality, determined by two-sided log-rank test, and univariate Cox regression (HR, 0.21; 95% confidence interval [CI], 0.113-0.389; P = .00327).

Supplementary Table 1 shRNA sequence information

	**shRNA sequences**	**Clone ID**	**Facility**
CT26-scr	CCGGCCTAAGGTTAAGTCGCCCTCGCTCGAGCGAGGGCGACTTAACCTTAGGTTTTT	ASN0000000004	National RNAi Core Facility (Academia Sinica)
shppp2r1a-1 (mouse)	CCGGGCCACCAGCAACCTTAAGAAACTCGAGTTTCTTAAGGTTGCTGGTGGCTTTTT	TRCN0000012626	National RNAi Core Facility (Academia Sinica)
shppp2r1a-2 (mouse)	CCGGGCACCGAATGACTACACTCTTCTCGAGAAGAGTGTAGTCATTCGGTGCTTTTT	TRCN0000012624	National RNAi Core Facility (Academia Sinica)
shppp2r1a-1 (human)	CCGGACTGGATCCTGCTGCTGTAATCTCGAGATTACAGCAGCAGGATCCAGTTTTTG	TRCN0000231600	National RNAi Core Facility (Academia Sinica)
shppp2r1a-2 (human)	CCGGTTGCCAATGTCCGCTTCAATGCTCGAGCATTGAAGCGGACATTGGCAATTTTTG	TRCN0000231509	National RNAi Core Facility (Academia Sinica)
shRb1 (mouse)	CCGGTACCAGTACCAAGGTTGATAACTCGAGTTATCAACCTTGGTACTGGTATTTTTG	TRCN0000218613	National RNAi Core Facility (Academia Sinica)
shHDAC2 (mouse)	CCGGCGAGCATCAGACAAACGGATACTCGAGTATCCGTTTGTCTGATGCTCGTTTTTG	TRCN0000039397	National RNAi Core Facility (Academia Sinica)

Supplementary Table 2. RNA seq and gene set enrichment analysis. *Lgr5-EGFP-CreERT2; Ppp2r1a$^{flox/flox}$* intestinal organoids were treated without (control) or with DMBA or/and tamoxifen (TAM) for 50 days in the presence of EGF, Noggin and R-spondin 1. Gene set enrichment analysis (GSEA) shows "E2F" for the organoids treated with DMBA and TAM group versus control group, $p < 0.05$. Biological processes were shown with Normalized enrichment score (NES), False discovery rate (FDR) and Familywise-error rate (FWER) determined by GSEA.

NAME	NES	FDR q-val	FWER p-val
Oxidative_Phosphorylation	-0.69	0	0
Fatty_Acid_Metabolism	-0.5	0	0
E2f_Targets	-0.43	0	0
Epithelial_Mesenchymal_Transition	0.73	0	0
Il6_Jak_Stat3_Signaling	0.59	0	0
Angiogenesis	0.66	0	0
Inflammatory_Response	0.51	0	0
Coagulation	0.53	0	0
Kras_Signaling_Up	0.5	0	0
Tnfa_Signaling_Via_Nfkb	0.49	0	0
Interferon_Alpha_Response	-0.47	0	0.001
Tgf_Beta_Signaling	0.53	0.005	0.003
Complement	0.45	0	0.004
Il2_Stat5_Signaling	0.43	0	0.008
Hedgehog_Signaling	0.54	0.033	0.01
Myc_Targets_V1	-0.38	0	0.015
Hypoxia	0.41	0.005	0.017
Mtorc1_Signaling	-0.36	0	0.023
Estrogen_Response_Late	-0.34	0	0.028
Apoptosis	0.4	0.014	0.036
Notch_Signaling	0.51	0.084	0.039
Estrogen_Response_Early	-0.33	0	0.04
Glycolysis	-0.33	0	0.042
G2M_Checkpoint	-0.33	0	0.043

Supplementary Table 3. Intersection molecules of PPP2R1A interaction protein (Supplementary Data 1) and RNA-sequencing (seq) analysis of *Lgr5-EGFP-CreERT2; Ppp2r1a$^{flox/flox}$* intestinal organoids with or without DMBA or/and tamoxifen (TAM) for 50 days

Gene Symbol	Accession	Description	Coverage [%]	MW [kDa]
abhd16a	Q9Z1Q2	Phosphatidylserine lipase ABHD16A	4	63
akt1	P31750	RAC-alpha serine/threonine-protein kinase	10	55.7
anxa5	P48036	Annexin A5	39	35.7
ap1m1	P35585	AP-1 complex subunit mu-1	31	48.5
arhgdia	Q99PT1	Rho GDP-dissociation inhibitor 1	42	23.4
bccip	Q9CWI3	BRCA2 and CDKN1A-interacting protein	3	35.9
c3	P01027	Complement C3	4	186.4
calr	P14211	Calreticulin	38	48
cand1	Q6ZQ38	Cullin-associated NEDD8-dissociated protein 1	2	136.2
capza1	P47753	F-actin-capping protein subunit alpha-1	35	32.9
clta	Q3THU7	Clathrin light chain	25	23.5
cnp	P63017	Heat shock cognate 71 kDa protein	54	70.8
col1a1	P11087	Collagen alpha-1(I) chain	1	137.9
csrp1	P97315	Cysteine and glycine-rich protein 1	65	20.6
des	P31001	Desmin	49	53.5
eef1a1	P10126	Elongation factor 1-alpha 1	61	50.1
eif2s1	Q6ZWX6	Eukaryotic translation initiation factor 2 subunit 1	34	36.1
faf2	Q3TDN2	FAS-associated factor 2	24	52.4
gdi1	P50396	Rab GDP dissociation inhibitor alpha	45	50.5
h2-ab1	P06342	H-2 class II histocompatibility antigen, A-Q beta chain	6	29.9
hdac2	A0A0R4J008	Histone deacetylase 2	12	55.3
hspa5	P20029	Endoplasmic reticulum chaperone BiP	38	72.4
ilkap	Q8R0F6	Integrin-linked kinase-associated serine/threonine phosphatase 2C	6	42.7
inpp1	P49442	Inositol polyphosphate 1-phosphatase	3	43.3
kcmf1	Q80UY2	E3 ubiquitin-protein ligase KCMF1	4	41.8
lgals1	P16045	Galectin-1	15	14.9
mapk14	P47811	Mitogen-activated protein kinase 14	12	41.3
msn	P26041	Moesin	37	67.7
myh10	Q3UH59	Myosin-10	3	233.3
nagk	Q9D997	N-acetyl-D-glucosamine kinase	8	39.3
nfkb1	P25799	Nuclear factor NF-kappa-B p105 subunit	1	105.5
ostf1	Q62422	Osteoclast-stimulating factor 1	22	23.8
pacsin3	Q99JB8	Protein kinase C and casein kinase II substrate protein 3	17	48.6
pak2	Q8CIN4	Serine/threonine-protein kinase PAK 2	26	57.9
pgm2	Q66JR7	Pgm2 protein (Fragment)	56	64.1
phyhip	Q8BGT8	Phytanoyl-CoA hydroxylase-interacting protein-like OS=Mus musculus OX=10090 GN=Phyhipl PE=1 SV=1	7	42.3
pld3	O35405	Phospholipase D3	7	54.4
plin1	Q8CGN5	Perilipin-1	6	55.6
ppme1	Q8BVQ5	Protein phosphatase methylesterase 1	12	42.2
ppp1ca	P62137	Serine/threonine-protein phosphatase PP1-alpha catalytic subunit	41	37.5
ppp2cb	P62715	Serine/threonine-protein phosphatase 2A catalytic subunit beta isoform	38	35.6
ppp2r2a	Q6P1F6	Serine/threonine-protein phosphatase 2A 55 kDa regulatory subunit B alpha isoform	13	51.7
ppp2r2d	Q925E7	Serine/threonine-protein phosphatase 2A 55 kDa regulatory subunit B delta isoform	14	51.9
ppp2r5e	Q61151	Serine/threonine-protein phosphatase 2A 56 kDa regulatory subunit epsilon isoform	3	54.7
ppp3ca	P63328	Serine/threonine-protein phosphatase 2B catalytic subunit alpha isoform	20	58.6
ppt1	Q8VBX5	Palmitoyl-protein thioesterase	7	34.6
prkaa1	Q5EG47	5'-AMP-activated protein kinase catalytic subunit alpha-1	5	63.9
prkaca	P05132	cAMP-dependent protein kinase catalytic subunit alpha	27	40.5
psat1	Q3ULZ3	Phosphoserine aminotransferase	11	40.4
rabgef1	Q9JM13	Rab5 GDP/GTP exchange factor	2	56.8
rad23b	P54728	UV excision repair protein RAD23 homolog B	21	43.5
rb1	P06400	Retinoblastoma protein	18	38.1
sarnp	Q9D1J3	SAP domain-containing ribonucleoprotein	23	23.5
strap	Q9Z1Z2	Serine-threonine kinase receptor-associated protein	39	38.4
taf1	D3YZK4	Transcription initiation factor TFIID subunit	0	215.7
tuba1a	P68369	Tubulin alpha-1A chain	83	50.1
u2af2	P26369	Splicing factor U2AF 65 kDa subunit	21	53.5
ywhab	Q9CQV8	14-3-3 protein beta/alpha	66	28.1

Supplementary Table 4. The gene set enrichment analysis of 58 proteins identified in Supplementary Table 3 is shown. Pathways of functional gene sets are evaluated by input gene lists with determined by Hypergeometric test and Fisher's exact test[1] .

Pathway analysis	Input number	Corrected P-Value	Input
Regulation of TP53 Activity	8	4.10E-08	Hdac2, taf1, ppp2cb, prkaa1, strap, mapk14, akt1, rb1
Cell Cycle	11	1.02E-06	Ppp2r2d, tuba1a, ppp2cb, ppp2r2a, rb1 akt1, ppme1, prkaca, ywhab, Hdac2, ppp2r5e
RNA Polymerase II Transcription	12	4.07E-06	Hdac2, u2af2, taf1, ppp2cb, prkaa1, strap, mapk14, akt1, sarnp, col1a1, ywhab, rb1
Signaling by WNT	6	3.81E-05	Ppp2cb, akt1, prkaca, clta, ppp3ca, ppp2r5e
Post-translational protein modification	10	4.47E-05	Capza1, hdac2, rad23b, cand1, eef1a1, calr, tuba1a, c3, lgals1, nagk
Proteoglycans in cancer	5	6.32E-05	Mapk14, msn, akt1, prkaca, ppp1ca
PD-L1 expression and PD-1 checkpoint pathway in cancer	4	6.75E-05	Mapk14, ppp3ca, nfkb1, akt1
Cyclin A/B1/B2 associated events during G2/M transition	3	7.90E-05	Ppme1, ppp2cb, ppp2r2a
T cell activation	3	0.000973	Ppp3ca, nfkb1, akt1
Toll Like Receptor Cascade	3	0.001191	Mapk14, nfkb1, ppp2cb
Beta-catenin phosphorylation cascade	2	0.001521	Ppp2r5e, ppp2cb
Mitotic G1-G1/S phases	3	0.00349	Akt1, ppp2cb, ppp2r2a

Supplementary Table 5. Putative neoantigens, shown by sequence and gene symbol, ranked by probability for the CT26-Shppp2r1a sample

Rank	MHC	Sequence	Gene	probability	FPKM	NetH2pan	MHCflurry 2.0
1	H-2-Kd	LTLNKSSSTAYMELR	Ighv1-22	1	1.037	468.4	485.88
2	H-2-Kd	NYRSSGCSAVSAETA	Cep44	1	1.338	468.61	626.89
3	H-2-Kd	APSTTHILSTTSSTQ	Muc4	1	0.74	473.21	349.95
4	H-2-Kd	SQPFRLTCTFSGFSL	Ighv8-4	1	1.229	476.45	585.78
5	H-2-Kd	FNAAGFFHAHISYLE	Mon1a	1	0.908	479.46	435.34
6	H-2-Kd	AFENGRVLVELSGNS	Gmip	1	1.128	480.08	541.5
7	H-2-Kd	SFEEIIADAILNNRI	Spg11	1	1.1	480.68	528.51
8	H-2-Kd	GSGRQFSLKISSLHP	Igkv12-89	1	1.068	481.71	514.57
9	H-2-Kd	FLFLLSVTTGNGETS	Ighv1-12	1	1.085	483.54	524.41
10	H-2-Kd	LAPSICVAPYPSARS	Vars2	1	1.343	487.78	655.22
11	H-2-Kd	NPGIKLQVAPIPRIC	Ckap2l	1	0.742	488.79	362.45
12	H-2-Kd	NNYATHYAESVKGRF	Ighv6-3	1	1.022	490.41	501.24
13	H-2-Kd	LLLLIVPAYVLSQVT	Ighv8-12	1	0.949	490.63	465.61
14	H-2-Kd	CRSGFGVVHVADAVF	Trmt61a	1	2.007	491.67	986.66
15	H-2-Kd	TVLAVLGVASRFLNE	Mgst2	1	0.961	492.46	473.19
16	H-2-Kd	AKSLEWIGVISTYHG	Ighv1-67	1	1.631	492.57	803.2
17	H-2-Kd	HKSHTLIRGFQLFIK	Spg11	1	0.911	493.24	449.42
18	H-2-Kd	IAQRGGWVAALNLRR	Bak1	1	1.094	493.42	539.95
19	H-2-Kd	AKNTLYLQMSSLGSE	Ighv5-9	1	0.601	493.52	296.75
20	H-2-Kd	AMETRGMEARGLEMR	Cstf2t	1	1.87	493.63	923.28
21	H-2-Kd	WQEEQDVAAVVGKHA	Mia3	1	0.935	495.02	462.92
22	H-2-Kd	VQPSQSLSIICTVSG	Ighv2-5	1	1.296	495.06	641.79
23	H-2-Kd	LVPGTMCSPALAVSQ	Telo2	1	1.273	495.7	630.9
24	H-2-Kd	DRNTTVSATSSTLTS	Muc4	1	0.704	497.44	350.25
25	H-2-Kd	FTGLKNTESYAIKSV	Rif1	1	0.919	497.44	457.38
26	H-2-Kd	KLKEMAKAEGLWNLF	Acad11	1	1.385	499.13	691.06
27	H-2-Kd	RKMAELMLLSEIADP	Atf6b	0.923076923	1.113	432.38	481.26
28	H-2-Kd	EIPSVYSSVILGIKD	Ints8	0.923076923	1.003	433.26	434.38
29	H-2-Kd	SSSTAYMQLSSLTSD	Ighv1-54	0.923076923	0.894	433.29	387.43
30	H-2-Kd	KKRETEAKLMVANKP	Snx2	0.923076923	1.026	433.52	444.83

Supplementary Table 6. Distribution of the 20 most-frequent TCR rearrangements identified in peripheral blood from mice

CT26-control 1	Clone Fraction	All Chits With Score	CT26-control 2	Clone Fraction	All Chits With Score	CT26-control 3	Clone Fraction	All Chits With Score	CT-26-shppp2r1a 1	Clone Fraction	All Chits With Score	CT-26-shppp2r1a 2	Clone Fraction	All Chits With Score	CT-26-shppp2r1a 3	Clone Fraction	All Chits With Score
228	0.004684	TRBC2*00 (110.4)	142	0.00108	TRBC2*00 (111)	215	0.001635	TRAC*00 (142.8)	1638	0.010393	TRAC*00 (144.5)	838	0.006372	TRBC2*00 (111.1)	2452	0.016998	TRAC*00 (143.7)
203	0.00417	TRBC2*00 (111.8)	142	0.001034	TRAC*00 (143.4)	208	0.001582	TRBC2*00 (110.9)	1147	0.007278	TRAC*00 (144.9)	807	0.006137	TRBC2*00 (110.2)	1566	0.010856	TRAC*00 (143.6)
188	0.003862	TRBC2*00 (110.7)	136	0.001019	TRBC2*00 (109.5)	208	0.001582	TRBC2*00 (109.3)	691	0.004384	TRAC*00 (143)	796	0.006053	TRBC2*00 (111)	1061	0.007355	TRAC*00 (143.2)
148	0.00304	TRBC2*00 (110.3)	134	0.001004	TRBC2*00 (109)	206	0.001566	TRBC2*00 (108.8)	521	0.003306	TRAC*00 (143.9)	452	0.003437	TRBC2*00 (110.8)	687	0.004762	TRAC*00 (144.2)
146	0.002999	TRBC2*00 (111)	132	0.000989	TRBC2*00 (109.3)	194	0.001475	TRAC*00 (145)	272	0.001726	TRAC*00 (143.9)	382	0.002905	TRAC*00 (143.9)	636	0.004409	TRAC*00 (143.1)
145	0.002979	TRBC2*00 (111.6)	130	0.000981	TRBC2*00 (112.1)	191	0.001452	TRAC*00 (144.2)	174	0.001104	TRAC*00 (142.7)	357	0.002715	TRBC2*00 (111.2)	513	0.003556	TRBC2*00 (111.2)
130	0.002671	TRAC*00 (141.7)	129	0.000973	TRBC2*00 (112.6)	189	0.001437	TRAC*00 (142.9)	129	0.000818	TRBC2*00 (109.1)	351	0.002669	TRBC2*00 (110.7)	446	0.003092	TRBC2*00 (112.1)
128	0.002629	TRAC*00 (144.2)	128	0.000973	TRAC*00 (143.9)	187	0.001422	TRAC*00 (145.5)	105	0.000666	TRAC*00 (142.9)	347	0.002639	TRBC2*00 (109.6)	429	0.002974	TRBC2*00 (111.1)
119	0.002445	TRBC2*00 (112.2)	128	0.000943	TRAC*00 (143.5)	182	0.001384	TRBC2*00 (112.3)	88	0.000558	TRAC*00 (145.2)	333	0.002532	TRAC*00 (142.2)	320	0.002218	TRBC2*00 (111.3)
119	0.002445	TRBC2*00 (109.5)	124	0.000935	TRBC2*00 (111.6)	167	0.00127	TRAC*00 (144.3)	86	0.000546	TRAC*00 (145.9)	311	0.002365	TRBC2*00 (110.3)	266	0.001844	TRAC*00 (143)
110	0.00226	TRBC2*00 (109.8)	123	0.000935	TRAC*00 (146.4)	164	0.001247	TRBC2*00 (110.9)	85	0.000539	TRAC*00 (144.5)	302	0.002296	TRBC2*00 (111.8)	263	0.001823	TRBC2*00 (110)
105	0.002157	TRBC2*00 (112.1)	123	0.000928	TRAC*00 (144.7)	164	0.001247	TRBC2*00 (111.2)	84	0.000533	TRBC2*00 (114.8)	297	0.002258	TRBC2*00 (111.8)	237	0.001643	TRBC2*00 (109.7)
103	0.002116	TRBC2*00 (110.2)	122	0.000928	TRBC2*00 (110.7)	161	0.001224	TRAC*00 (144.4)	65	0.000412	TRBC2*00 (110.9)	275	0.002091	TRAC*00 (143.5)	215	0.00149	TRAC*00 (144.3)
102	0.002095	TRBC2*00 (111.4)	122	0.00092	TRAC*00 (145.3)	161	0.001224	TRBC2*00 (111.8)	63	0.0004	TRBC2*00 (113.1)	266	0.002023	TRBC2*00 (111.9)	211	0.001463	TRBC2*00 (111.4)
96	0.001972	TRAC*00 (143.6)	121	0.000905	TRBC2*00 (108)	161	0.001224	TRBC2*00 (113.5)	50	0.000317	TRBC2*00 (115.1)	264	0.002008	TRAC*00 (144.1)	201	0.001393	TRBC2*00 (111.4)
93	0.00191	TRBC2*00 (110.6)	119	0.000905	TRBC2*00 (111.8)	157	0.001194	TRBC2*00 (111.3)	48	0.000305	TRAC*00 (143)	249	0.001893	TRBC2*00 (110.6)	168	0.001165	TRBC2*00 (109.7)
92	0.00189	TRBC2*00 (113)	119	0.000897	TRAC*00 (143.3)	150	0.001141	TRAC*00 (144.3)	47	0.000298	TRAC*00 (142)	235	0.001787	TRAC*00 (143.7)	165	0.001144	TRBC2*00 (110.5)
90	0.001849	TRBC2*00 (110.8)	118	0.000859	TRBC2*00 (112.5)	150	0.001141	TRAC*00 (143.9)	46	0.000292	TRAC*00 (139.7)	226	0.001719	TRBC2*00 (111)	160	0.001109	TRBC2*00 (110.9)
88	0.001808	TRAC*00 (142.1)	113	0.000836	TRBC2*00 (110.7)	149	0.001133	TRAC*00 (145)	45	0.000286	TRAC*00 (143.6)	218	0.001658	TRBC2*00 (110.3)	153	0.001061	TRAC*00 (145)

Supplementary Table 7. Human tissue array containing MSI and MSS colorectal tumours was assayed for key protein levels by immunohistochemistry. The data were quantified using arbitrary grades for diaminobenzidine (DAB)-positive immunostaining patterns.

Severity	Grade
*NR (<1%)	0
Minimal (1~5%)	1
Mild (5~25%)	2
Moderate (26~50%)	3
Moderate high (51~75%)	4
Severe high (>75%)	5

Supplementary Reference:

1 Bu, D. *et al*. KOBAS-i: intelligent prioritization and exploratory visualization of biological functions for gene enrichment analysis. *Nucleic Acids Res* **49**, W317-W325, doi:10.1093/nar/gkab447 (2021).
