# Supplementary material for: Protein phosphatase 2A inactivation induces microsatellite instability, neoantigen production and immune response
Source: Nat Commun. 2021 Dec 15;12:7297. doi: 10.1038/s41467-021-27620-x (PMC8674339; doi:10.1038/s41467-021-27620-x)
Supplement: Supplementary file 3 — Description of Additional Supplementary Files [file 41467_2021_27620_MOESM3_ESM.pdf]

File Name: Supplementary Data 1. PPP2R1A-interacting proteins

Description: Mass spectrometry data of PPP2R1A-interacting proteins from normal intestinal organoid cultures.
